# Bone marrow granulomas in a high tuberculosis prevalence setting: A clinicopathological study of 110 cases

**DOI:** 10.1097/MD.0000000000009726

**Published:** 2018-01-26

**Authors:** Yu Wang, Xiao-Yan Tang, Ji Yuan, Shou-Quan Wu, Guo Chen, Miao-Miao Zhang, Ming-Gui Wang, Wen-Yan Zhang, Jian-Qing He

**Affiliations:** a Department of Respiratory and Critical Care Medicine, West China Hospital, Sichuan University, Chengdu, People's Republic of China; b Department of Pathology and Microbiology, University of Nebraska Medical Center, Omaha, NE, USA; c Department of Pathology, West China Hospital, Sichuan University, Chengdu, People's Republic of China.

**Keywords:** biopsy, bone marrow, granulomas, prevalence, tuberculosis

## Abstract

Granulomas were reported in 0.3% to 3% of bone marrow biopsies. The aim of the study was to evaluate the incidence and etiology of bone marrow granulomas (BMGs) in the West China Hospital, which located at a high tuberculosis (TB) prevalence area in China.

A retrospective case review was performed on 11,339 bone marrow biopsies at the West China Hospital of Sichuan University between January 2011 and December 2015. Cases with BMGs were retrieved and their clinical data and histopathological features were collected, examined, and analyzed.

Out of 11,339, 110 cases showed granulomatous lesions in the bone marrow biopsies (0.97%). Etiologies were indentified in 80 cases (72.8%), with infections being the most common (64.5%), following by malignancies (4.5%) and autoimmune diseases (3.6%). Among infectious cases, 87.32% (62/71) cases were diagnosed as TB, a positive acid-fast stain or/and polymerase chain reaction (PCR) result for mycobacterium TB DNA fragment amplification was obtained for 35 cases. In 30 cases (27.27%), a definite diagnosis could not be established.

In a TB high prevalence region in China, with a combined histological, clinical, serological, and molecular approach, we were able to clarify the cause in 72.73% of the bone marrow granulomatous cases. TB is the most common underlying etiologies. Therefore, acid-fast stain and quantitative PCR for mycobacterium TB DNA amplification are recommended as a routine for bone marrow biopsies in TB high prevalence regions.

## 1 Introduction

Granuloma is defined as the accumulation of macrophages, epithelioid cells, and other inflammatory cells with or without giant cells.^[1]^ Different types of granulomas have been used, such as sarcoidal, tuberculoid, palisaded, suppurative, and foreign body granuloma.^[2]^ They are useful in forming a differential diagnosis, but they are not absolute. For example, granuloma with caseous necrosis, commonly seen in tuberculosis (TB), can be also seen in fungal infection.^[3,4]^ Granuloma is a relative common finding of bone marrow biopsies and can be caused by a spectrum of underlying infectious and noninfectious disorders. Bone marrow granulomas (BMGs) caused by TB indicate an advanced stage with a high mortality in TB patients.^[5]^ The incidence of BMGs was reported range from 0.3% to 3% and TB accounted for less than half of the cases in the low TB prevalence regions or years.^[1]^ A series of 20 BMG cases by Feng et al^[6]^ from the Peking Union Medical College Hospital in China reported that 60% of BMGs were caused by TB.^[6]^ However, there is no large-scale study regarding incidence and etiologies of BMGs in the high TB prevalence region.

Our study evaluated the incidence and different etiologies of BMGs in the West China Hospital, one of the largest tertiary hospitals in China, which located in a high TB prevalence province. We also compared our results with the previous 7 studies of BMGs in the literatures.

## 2 Materials and methods

Ethical approval for the study was obtained from the Institutional Review Board of the West China Hospital of Sichuan University. A retrospective case review was performed among cases with bone marrow biopsies. Clinical and histopathological features were collected from the electronic files of the West China Hospital, in Sichuan province of China. The cases with granulomatous lesions found in the bone marrow biopsy from January 2011 to December 2015 were collected and reviewed by the pathologist (WYZ) to confirm the presence of granulomas. A literature review was also carried out to search for reports of case series with granulomatous lesions in the bone marrow and etiologies in each of the reports were compared with the present study.

The clinical data including demographic characteristics, medical history, presentation, and response to treatment were collected. The laboratory data of peripheral blood cell count, serum biochemistry, microbiology culture, and biopsy results from other sites, were also collected at the time of the initial diagnosis. An etiology of a disease was assigned based on the following: the definite etiology evidence was found by the same bone marrow specimen as which granulomas occurs, such as positive acid-fast stain and/or positive quantitative polymerase chain reaction (qPCR) results for mycobacterium TB DNA fragments or presence of malignant cells; the particular illness that known to cause granulomas was found in other organs or by the culture of other body sites; assigned with TB infections must have complete response to anti-TB treatment if no definite pathogen evidence was found; autoimmune diseases must have positive serological indexes, while infectious with virus must have positive serum lgA or lgM and positive PCR results; the common infectious diseases that can cause granulomas should been excluded if diagnosed as noninfectious diseases.

## 3 Results

### 3.1 Incidence of BMGs

Over the 5-year period of study between January 2011 and December 2015, 21,546,706 patients visited the West China Hospital. A total of 11,339 patients received bone marrow biopsies examination (52.6/100,000 patients), among which granulomas were found in 110 (0.97%), with an annual rate of 22 cases. The indications for marrow examination were fever of unknown origin (11 cases), hematological abnormality (14 cases), hepatosplenomegaly (2 cases), or combinations of above-mentioned symptoms/signs (74 cases).

### 3.2 Demographic and laboratory features

The demographic characteristics and clinical presentation and laboratory findings of the 110 cases are summarized in Table 1. Majority (82.7%) of these patients are Chinese Han. The most common presentation was fever (71.8%). Majority of patients had anemia, lymphocytopenia, and hypoalbuminemia.

**Table 1:**
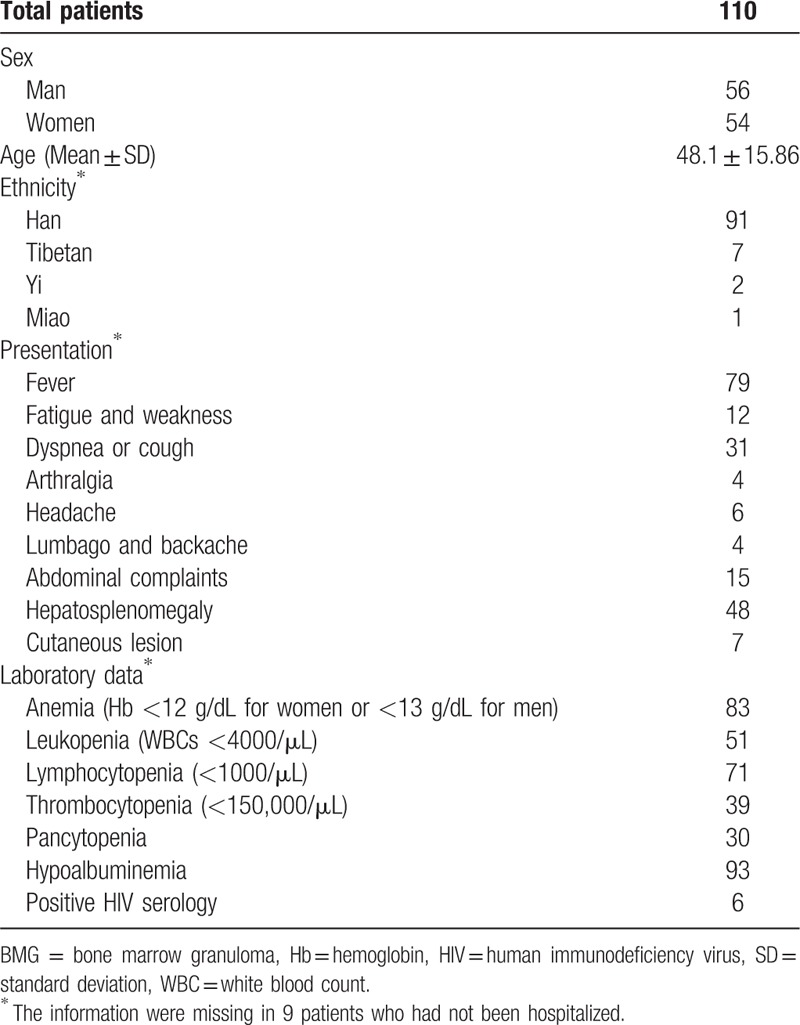
Demographic characteristics and laboratory data of patients with BMG.

### 3.3 Causes of BMGs

A total of 110 patients are divided into 6 major groups based on the standard description above (Table 2). In our study, infectious etiologies were found in 71 cases, 64.5% of total granulomatous cases. Among these 71 cases, 62 cases had TB infections, and the rest had fungal infection (4 cases), typhoid or paratyphoid fever (3 cases), and brucellosis (2 cases). Noninfectious etiologies were found in 9 cases, including 5 cases of hematological system diseases (non-Hodgkin lymphoma 3 cases and myelodysplastic syndrome 2 cases), and 4 cases of autoimmune diseases (1 case of Sjoegren syndrome, 1 case of antineutrophil cytoplasmic antibodies (ANCA)-associated vasculitis, 1 case of lupus, and 1 case of mixed connective tissue disease). A definitive etiology was not identified in 30 cases, accounted for 27.2% of all cases of BMGs. The previous studies of BMGs are also listed in Table 2. Compared with previous studies, present study has the highest percentage of infectious cases and TB takes the most part of it. This indicates that TB is the most common cause of BMGs in a region with high TB prevalence.

**Table 2:**
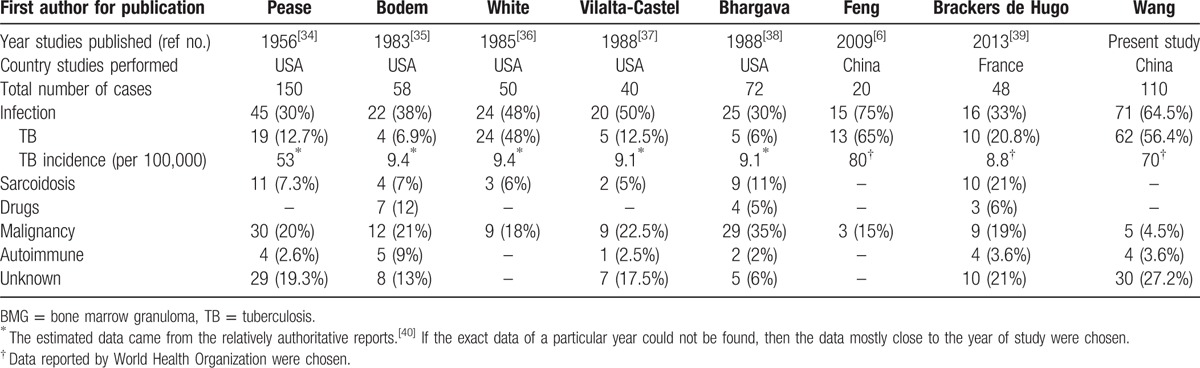
Comparison of etiologies of BMGs of previous major series with present study.

### 3.4 Infectious etiologies of BMGs

#### 3.4.1 Tuberculosis

During the 5 years from January 2011 to December 2015, there were a total of 55,441 patients diagnosed with TB in the West China Hospital based on the medical history, physical examination, test for TB infection (tuberculin skin test or QuantiFERON TB-GIT assay), chest radiograph, and microbiologic examination on sputum, urine, faeces, or other available body fluid. However, there are also 128 had undergone an examination of bone marrow biopsy due to an unexplainable fever, hematological abnormality, hepatosplenomegaly, or combination. We observed the TB-BMG in 62/128 cases (34 cases were men and 28 were women, ranging from 21 to 77 years of age). Thirty-five cases had a positive acid-fast stain and/or a positive qPCR result for tuberculosis bacilli (Table 3). Surprisingly, only 2 cases showed caseating granulomas and the rest had noncaseating granlomas. The diagnosis of TB in the remaining 27 cases was based on the clinical diagnosis and excellent responses to anti-TB drugs. The bone marrow and sputum were the most frequent sources where tuberculosis bacilli were found. All of these patients had inflammatory changes in computed tomography and 32 cases had diffuse small nodules in lung consistent with military TB.

**Table 3:**
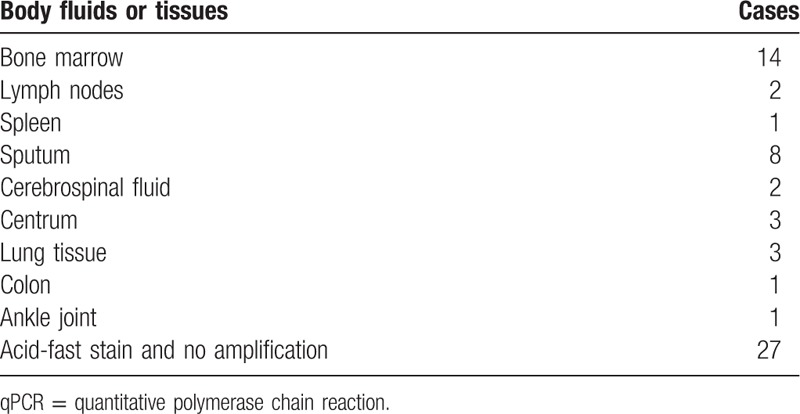
Positive acid-fast stain or qPCR for tuberculosis bacilli DNA segment in different body fluids or tissues.

The hypofunction of immune system was noticed in the TB-BMG patients. Six of these patients were diagnosed with acquired immune deficiency syndromes. Four patients were diagnosed with hepatocirrhosis, including 3 cases of hepatitis B virus-related cirrhosis and 1 case of long-term alcohol abuse-related cirrhosis. Five cases had a long history of using corticosteroid. The malignancies were found in 4 patients of TB-BMG. Three patients were diagnosis with type 2 diabetes mellitus. Therefore, the suppression of immune system and complication with underlying diseases might be the original or promote factors of TB-BMG. Most of the patients recovered with anti-TB treatment but unfortunately 6 of these individuals died after an average 19 of days’ hospitalization (range from 3 to 34 days). Among these patients, 4 cases had acute bleed due to thrombocytopenia and/or coagulopathy caused by TB in different body sites (2 cases in alimentary tract, 1 case in encephalic and the other 1 in peritoneal cavity).

#### 3.4.2 Typhoid fever and paratyphoid fever

Typhoid fever was present in 2 patients and paratyphoid fever in 1 patient. Blood cultures were positive in all cases. The 3 patients presented with the high temperature of >40°C. The decrease of peripheral whole blood cell count was found in these cases and hemophagocytosis was in 1 bone marrow biopsy. Examination of the stool was performed for 2 of them but pathogen could not be identified for both cases. One of these 2 patients and her family had a history of severe diarrhea after a family dinner. Their body temperature soon returned to normal and the peripheral blood count improved with 1 course of antibiotic treatment.

#### 3.4.3 Fungal infections

Four patients had fungal infection. The fungus *Histoplasma capsulatum* was found in the macrophages in both the biopsy of bone marrow and colonic mucosa by periodic acid Schiff stain in 1 case. Two men and 1 woman were diagnosed with other fungal infection (2 were candidiasis and 1 was *Penicilliosis marneffei*), all of whom had a human immunodeficiency virus (HIV) coinfection. The pathogen was found in cultures from oral cavity and esophagus mucosal secretion and/or bone marrow cultures. Clinical symptoms and sign of all cases were improved soon after antifungal therapy.

#### 3.4.4 Brucellosis

Two patients had brucellosis infection. One patient was a 21-year-old student. She had a chief complain of fever >1 month. Brucella spp was found in blood cultures. The second patient was a 48-year-old pasturer. He had persistent fever for 6 months and had been diagnosed with alcoholic cirrhosis due to a long-term alcohol abuse. Brucella spp was found by bone marrow culture. He had a co-infection of Epstein–Barr virus and herpes virus, which was diagnosed with positive serum antibody and DNA amplification. The symptom improved soon with application of minocycline and rifampicin.

### 3.5 Noninfection etiologies of BMGs

#### 3.5.1 Hematological system diseases

Three women and 2 men had hematological malignancies (2 with non-Hodgkin lymphoma of T-cell origin and 1 with natural killer (NK)/T-cell lymphoma and 2 with myelodyplastic syndrome). They aged from 31 to 78. Diagnosis was based on histological examination and immunophenotyping of bone marrow, lymph nodes, or skin. Hemophagocytosis was seen in a bone marrow biopsy of the patient diagnosed with NK/T-cell lymphoma and he died of serious infections 20 days after admission. In addition, the serum antibody of Epstein–Barr virus (immunoglobulin A) was positive in 1 patient with myelodyplastic syndrome and the patient also died of infection 13 days after hospitalization.

#### 3.5.2 Autoimmune diseases

Four cases of autoimmune disease were incorporated in our study. Three women aged from 23 to 36 were diagnosed with systemic lupus erythematosus, Sjogren's syndrome, and mixed connective tissue disease respectively by the appearance of characteristic serum antibodies and clinical symptoms. ANCA was positive in one 77 year-old man who complicated with chronic renal failure.

### 3.6 Unknown etiologies

Among the 110 cases we reviewed, 69 cases had an initial clinical diagnosis which was further verified by bone marrow biopsy and 11 cases were diagnosed by bone marrow biopsy. Unfortunately there were 30 patients whose final diagnoses were either unknown or could not be reasonably associated with granuloma formation. Among these patients, 2 patients died before a diagnosis could be made and autopsies were rejected. Thirteen patients refused further examination and treatment.

## 4 Discussion

Making an exactly etiological diagnosis is essential in clinical strategy. In general, noninvasive samples which are easy to access, for example, saliva, sputum, serum, plasma, blood, urine, tissue, fecal water, or feces, commonly meet the need in diagnosis with most diseases.^[7]^ In the present study, a total of 55,441 patients were diagnosed with TB with merely 128 undergone a bone marrow biopsy. However, a bone marrow biopsy is also feasible and even needful on some occasions, such as the fever of unknown origin or hematological abnormality, to identify an underlying disorder.^[8,9]^ We observed an incidence of 62/128 patients had an finding of TB-BMGs and a high risk for poor outcome (6/62 deaths within an average 19 of days), suggesting that a bone marrow biopsy should be taken into consideration for patients with such situations.

We systemically reviewed and summaried the etiologies with a rare pathological findings in marrow biopsy—granuloma. However, the mechanism of granuloma formation is very complex, which contains a series of interactions of pathogen, cytokines, and inflammatory cells.^[10,11]^ Many disorders have been implicated in the pathogenesis of BMG. They mainly encompass infectious diseases, autoimmune diseases, drugs, malignant lesions, and sarcoidosis. Although many laboratory approaches have been established to unveil the mysteries of the complex relationships taking place in the granuloma,^[10]^ the diagnosis cannot be made solely on pathological criteria and there also have some cases remain unknown, for the unspecified morphological feature of bone marrow and no characteristic symptom. There are also some reports that fibrin ring granulomas may be a special feature of Epstein–Barr virus or *Rickettsia typhi* and Doughnut ring-shaped epithelioid granulomas for Q-fever,^[12–14]^ but it also need more observation and validation.

For the complex individual variations in clinical presentations and various etiologies, detail epidemiological evidence is needed to help clinical decision. But a large-scale study of BMG, especially in the region with high burden of TB, is lacking. Comparing with the 7 previous major serial studies of granulomas, the present study had high percentage of fever (79%) and hepatosplenomegaly (48%). The laboratory data had no remarkable difference. Anemia, leukopenia, thrombocytopenia, pancytopenia, and hypoalbuminemia could be easily found among these patients.

Sichuan province is a TB high-burden region where the prevalence of TB is between 549 per 100,000 and 1656 per 100,000.^[15]^ TB-BMG accounted for a larger proportion among the various etiologies of BMG in this TB high epidemic area.^[16]^ We reported that 56.3% (62/112) of BMGs were due to TB, which was closed to the incidence of 65.0% reported by the researchers from Peking Union Medical College Hospital (13/20).^[6]^ While in the TB low epidemic area or years (mandatory defined as incidence of TB <50 per 100,000 population in this study), such as in USA or in France (shown in Table 2), TB-MBG is less common (17.9%, 48/268) comparing with that in the TB high epidemic area or years (33.6%, 94/280). Therefore, TB should be considered first in the TB high epidemic region if BMGs were found.

It is still debating whether a bone marrow biopsy should been performed in disseminated TB.^[17,18]^ However, a bone marrow biopsy remains an important examination in the patients of fever of unknown origin,^[19]^ especially in immunocompromised patients.^[20]^ Tajiri et al^[21]^ reported that in autopsy cases of miliary TB, 86% had histological features of nonreactive exudative inflammation, along with granulomas containing giant cells with or without caseous necrosis in the bone marrow. Military TB with abnormal peripheral blood counts suggested a high chance of BMG secondary to TB.^[22]^ If the granulomatous lesion is seen in the bone marrow biopsy, it provides an effective proof of TB infection.

In the present study, 14/62 had microbiological evidence of TB in bone marrow where acid-fast bacilli and/or TB-DNA qPCR detection was positive. Direct microbiological evidence of TB can also be found in sputum, cerebrospinal fluid, lung, and other tissue biopsies in 21 cases. However, 27 cases remained no direct microbiological evidence of TB but had excellent responses to anti-TB therapy after excluding other possible diseases. In addition, anti-TB therapy should be administered urgently to prevent an otherwise fatal outcome in military TB,^[23]^ therefore, a combined histopathological, clinical, and serological approach is recommended to render an early diagnosis. It should also be noted that a part of these patients with granulomatous lesion in bone marrow had chronic diseases, AIDS, or long-term use of corticosteroid for different reasons. This may promote the infection and dissemination of TB for suppression of immune function.^[24]^

Typhoid fever is known to induce granuloma formation in many organs, such as digestive tract, liver, spleen, and bone marrow.^[25]^ The primary affected organ is gastrointestine.^[26]^ Interestingly, salmonella was found not by the examination of stool but blood culture in all the cases in our study. It may due to the deferred inspection of stool. Histoplasmosis usually occurs in the immunocompromised individuals.^[27]^ For the immunocompetent subjects, the chance of histoplasmosis is 1 per 2000 and the typical tissues involved are central nervous system, liver, spleen, and rheumatologic, ocular, and hematologic system.^[28]^ The patient diagnosed with histoplasmosis in our study had no coinfection with HIV or immunosuppressive disorders and the pathogen was found in bone marrow and colon. Diagnosis of brucellosis requires isolation in blood culture and/or bone marrow,^[29,30]^ or serological evidence,^[31]^ or enzyme linked immunosorbent assay.^[32]^ Both cases in our study had positive blood cultures of brucellosis. Therefore, blood culture is an important approach that can help to make correct diagnosis if specific pathogen infection is suspected.

A wide range of autoimmune diseases can be found including ANCA-associated systemic vasculitis, systemic lupus erythematosus, Sjögren's disease, and mixed connective tissue diseases in our study. Numerous solid tumors have been described in association with BMG especially Hodgkin lymphoma and non-Hodgkin lymphoma.^[33]^ In our BMG cases, only non-Hodgkin lymphoma can be found and they were of respectively T-cell origin and NK/T-cell origin by the flow cytometry assay. However, sarcoidosis and drug-induced BMG were not presented in the present study.

We could not identify any underlying diseases in 30 patients, which accounted for 27.3% of all cases. The percentage of unknown etiologies is higher than previous reported studies. We reasoned that not only the complicated pathogenesis and lack of awareness of these diseases but also the low economic conditions limited further diagnosis and treatment.

In conclusion, our findings showed that a definite etiology can be established in the majority of cases with BMGs and this need a comprehensive approach including histopathological, clinical, serological, and molecular examinations. In a TB high prevalence region in China, TB is the most common underlying etiology. Therefore, acid-fast stain and qPCR for mycobacterium TB DNA amplification are recommended as a routine for bone marrow biopsies in TB high prevalence regions.

## Acknowledgments

The authors thank the information Center of West China hospital for assistance in obtaining and reviewing the pathological sections and clinical information.
